# Removal of an Intestinal Stricture

**Published:** 1855-03

**Authors:** 


### MEDICINE IN HOLLAND.

*Removal of an intestinal stricture.* By Dr. Van Dommelen.—P., aged 29, of a choleric temperament, a soldier, in the 6th Regiment of Infantry, and formerly in the East India army, was attacked at the end of 1849, at Soerabaya, with dysentery, for which he was for three months under treatment in the Hospital of that place, and from which he recovered, but has since had extreme difficulty in passing fæces. In 1851, he was, according to his statement, set home on account of the constant recurrence of palpitations of the heart, which the Physicians attributed to the heat being more than he could endure. He remained at home as a citizen for a year, when he got himself received into the Militia; but from the time he joined he was repeatedly admitted into Hospital, complaining of colicky pains. The peculiar appearance of the fæces immediately struck us; they were, in fact, not thicker than the stem of a pipe, and long, like a small cord in the bed-pan. This was readily explained, on local examination; the finger introduced into the rectum encountered, at a distance of eight centimetress, (3·1496 English inches,) from the anus, a ring-shaped septum, which did not admit the passage of even the point of the pipe of an enema syringe. This septum was tolerably tense, and was as hard as cartilage, especially round the opening.

After having in vain endeavored, by means of evacuants, laxatives, and enemata, to regulate the alvine discharges, I proposed operation, to which the patient at once consented. Everything which could be necessary during its performance having been prepared, and the rectum having been previously cleared out, he was placed as for lithotomy, and was steadily held in this position by assistants. I now introduced the index-finger of the right hand, smeared with cerate, into the rectum, and so far through the stricture that the opening rested exactly on the top of the finger. I afterwards, with my left hand, passed a long, slender, blunt-pointed bistoury, the end of which was protected by having lint rolled round it, along the index finger, to the stricture; I then drew the finger a little backwards behind the knob of the bistoury, by which means the latter came exactly opposite the narrow opening, and so passed into it. I now turned the back of the bistoury towards my finger, and with the latter pressed upwards, successively to both sides, and downwards. Immediately these incisions were made, the constriction ceased, and the finger, with the bistoury, with great ease, glided further up.

As there was no hæmorrhage of any consequence, no plugs were introduced, and the patient was merely recommended to keep perfectly quiet.

In the afternoon (seven hours after the operation) the patient passed some coagula of blood, mixed with serum, but without fæces. He also occasionally perceived a spasm in the sphincters, but was in other respects well. Early next morning he had a fæcal evacuation, differing in no respect either in form or quantity from what would be passed by the most healthy man. He continued under my observation during a period of three weeks, and I had each day an opportunity of observing the beneficial effects of the operation. I met him three months afterwards, when he stated that he had had no return of his infirmity.

From the distance of this stricture from the anus, we thought that a decisive result was to be expected only from such an operation as that above described. The introduction of plugs of lint, of little bags to be afterwards filled up with lint, or of bladders distended with air or fluid, would in this case have been inefficacious; the shortness of the canulas of Bermond de Bordeaux, which consist of cylinder over cylinder, and are only six centimetres (a little more than two and a-third English inches) in length, would prevent their being available; while, even if this were not the case, it would have been difficult, on account of the great exertion used in defecation, to retain the external cylinder in its position during the removal of the most internal necessary to admit of the discharge of that function; and, lastly, Costallat's instrument (a closed piece of intestine, introduced on a probe, and subsequently filled with lint) could scarcely have been introduced with the probe alone.

Dr. Van Munnikrede has described a case of medullary carcinoma of the femur—amputation—recurrence of the disease in the lungs—death. The patient was a farmer, aged 41. The carcinoma had been slowly developed, and was probably the result of an external injury, received many years before on the outer side of the knee. When amputation was first proposed, the tumour was painless, and had not yet become softened, and the general health was undisturbed. The patient refused to submit to the operation, nor did he consent to it until six months later, when the swelling having become larger and painful, presented here and there fluctuating points; the leg and foot were œdematous and hot; the general condition was impaired; hectic fever, perspiration, and general emaciation were present; the patient had a cachectic aspect. Three months after the performance of the operation, which took place under chloroform, he was released from treatment, in an improved state of health, the principal functions being duly performed. On examining the amputated limb, it was seen that the tumour was formed by an extraordinary enlargement of the lowest portion of the femur, and that it belonged to Nelaton's third variety of osseous cancer, the so-called "spina ventosa." About six months after the operation, the patient returned with an affection of the lungs, which was diagnosed to be carcinoma, and under which he sank in four months from the time of his return. On opening the thorax, the lungs were found to be throughout the greater part studded with cancerous nodules, some of which were hard, while others had passed into the stage of softening. Permission was not obtained to examine the other cavities of the body.

Dr. Muntendam gives a short statement of the clinical results obtained by him as to the use of quina in phthisis pulmonalis. The number of cases briefly reported by him amounts to 22; by the denomination "phthisical," he understands those who labor under pulmonary tubercles, who, at the same time, are feverish, and whose febrile condition is dependent on the morbid process in the lungs. He thinks that, from his observations, he may infer: 1st. That quina administered with, and often without, acetate of morphia, is capable, in very many cases, of prolonging the lives of phthisical patients, and that it not unfrequently even saves them, if fresh depositions of tubercle do not again excite the morbid condition. 2nd. It is capable of prolonging life where the local process is not too extensive; when administered in the commencement of the disease it may, especially in children, married and puerperal women, if circumstances are in other respects favorable, save life. 3rd. The frequency of the pulse does not diminish under the use of quina until later, after the assimilation is restored; or it may also do so in a person laboring under pulmonary tubercle, who has been attacked with intermittent fever. 4th. The transient hyperæmias or determinations to the head, chest, or intestinal canal in these patients, do not contraindicate the use of this remedy. The fever for the most part dissappears after some time, but is easily re-excited; or the febrile attacks may be obstinate, and may require the administration of quina to be continued for some weeks. In acute, and in many chronic cases, the cough and expectoration are at first increased; an indirect effect of the quina is also the recurrence of the menses; the effusion in the lungs remaining, as œdema, or hydrops-fibrinosus, after the congestions have disappeared, is then expectorated under the form of a gelatinous, more or less frothy mucous mass, or as serum, together with, in chronic cases, globose, nummular sputa. 5th. Dietl justly observes, that the effect of quina on the organs of the circulation is unexplained. 6th. Sulphate of quina administered continuously, and in small doeses, excites neither dyspnœa, nor diarrhœa, nor any other injurious symptom. 7th. Sulphate of quina deserves to occupy a principal place in the treatment of many, not to say of all cases of phthisis pulmonalis. 8th. Exclusion or antagonism between tuberculosis of the lungs and intermittent fever, does not exist, unless, perhaps, when by the latter term we understand "feverish dyscrasia."

Dr. Globee concludes his easay, "Something Further on Uræmia," in which he gives a sketch of the history of the group of phenomena comprehend under the term, with the communication of a case in which the functional symptoms of that condition and Bright's disease played a principal part. The patient, a man of 26 years of age, came under treatment with effusion in the left pleura, which had already lasted some weeks. He continued in the same state for above three months, after which time the case assumed all the appearance of uræmia. Although all the distinctive characters of the affection co-existed in all a well-marked degree, the presence of carbonate of ammonia could not be demonstrated either in the expired air, the blood, or any of the secretions. The patient sank in a fortnight from the time the first uræmic phenomena had appeared. On *post-mortem* examination, circumscribed arachnitis was observed on the upper surface of the brain; there was very great cerebro-spinal effusion; however, the author is of opinion, that uræmia must be looked on as the principal cause of death, when the condition of the kidneys, which were both found in the second stage of Bright's disease, is taken into account.—*London Medical Times and Gazette.*

